# Lithæmia

**Published:** 1885-08

**Authors:** James W. Putnam


THE BUFFALO MEDICAL AND SURGICAL JOURNAL

VOL. XXVI. AUGUST, 1885. No. 1.

### Original Communications.

### LITHÆMIA.

BY JAMES W. PUTNAM, M. D.*

The profession have so long been entertained by the recital of exciting hunts after bacteria micrococci and other minute forms of life, that it may be a pleasant change to study a disease which has not yet been accused of harboring even a microbe. In this attempt to prove the germ theory, and seeking in it the cause of most pathological conditions, the tendency has been to overlook and slight the chemical origin of disease and the changes induced by faulty nutrition of the blood.

The symptoms to which an impoverished or perverted blood supply may give rise are more tangible problems than the old conundrums: "Is the chicken the cause of the first egg, or is the egg the cause of the chicken, or is the bacillus the cause of the disease or its result?"

* Read before Buffalo Medical and Surgical Association.

In the treatment of disease by the advocates of the germ theory, the main endeavor is to find a germicide for that particular germ. This would be delightful therapeusis, if in so many cases the germ did not require a stronger lethal dose than does the patient.

The student and believer in the chemical origin of disease finds in lithæmia the study of perverted physiological conditions brought on by a disobedience of nature's laws of eating, drinking and exercise. He must deal with a disordered nutrition of the tissues caused by a blood supply which contains certain noxious principles. These noxious principles the physician attempts to remove by change of diet, regulation of exercise, and such medicines as we shall hereafter mention.

My attention was first drawn to this subject by an article published in the Boston *Medical and Surgical Journal*, Dec. 13, 1883, by Dr. G. H. Lyman, entitled, Tinnitus Aurium and Vertigo as Prominent Symptoms of Lithæmia. This article referred to Dr. DaCosta's paper on Nervous Symptoms of Lithæmia, published in the *American Journal of Medical Sciences*, Oct., 1881. From this the step was easy. Then Prout on Lithic Acid Diathesis, in 1843; Draper on Nature of the Gouty Vice, in '75, and Fothergill on Gout in its Protean Aspects; and Bulkley's essay on " The Gouty State in Diseases of the Skin."

Before speaking of Lithæmia as a distinct chronic disease, let me invite your attention to the words of Prout on the Pathology of the Formation of Lithic Acid:

" Lithic acid and its compounds we suppose to be principally derived from the albuminous principles, not only of the chyle and blood, but also of the albuminous textures of the body." When, on account of imperfect assimilation of alimentary matters by the stomach and primary assimilating processes, the chylous principles are not raised to that standard of perfection by which they are fitted to become component parts of the blood, he presumes the kidney has the power to separate them from the blood as Lithate of Ammonia.

Budd says: It was Dr. Prout who called our attention to this great class of disorders depending not on any visible structural change, but on some fault in those subtle processes that minister to nutrition. One of the most important of these is that condition in which there is an excess of lithic acid in the urine, indicating, Fothergill teaches us, "That certain peptones, instead of undergoing further elaboration, are turned aside and broken up prematurely into lithic acid and lithates. That these lithates do not represent tissue waste, for they have never been tissues." That is, to put it more plainly, that some of the food, which was swallowed for the purpose of digestion, absorption, and then assimilation failed somewhere in the chemical process, and instead of being assimilated and appropriated by the tissues, is lost so far as nutrition is concerned, and thrown off as lithic or uric acid.

Fothergill says: "The material which forms uric acid might have been tissue under more favorable circumstances."

Henry Burnett thinks that the presence of uric acid and the lithates is very much more frequently the result of defective digestion than of defective tissue metamorphosis, especially in dyspeptics.

Bulkley, after rehearsing the symptoms arising in lithæmics, says: "He who has gone somewhat more deeply into the subject recognizes that these are but the phenomena resulting from a blood alteration, which has been demonstrated, and that the presence of uric acid in the blood is the root of the evil, the attempted oxidation of which in the tissues gives rise to the local inflammations."

W. H. Draper, after drawing attention to the analogy of gout to glycosuria, says: Instead of being converted into the soluble and useless excrement urea, the oxidation of nitrogenous food is arrested at the formation of the penultimate uric or lithic acid, a very sparingly soluble substance, which, combining with the alkaline bases principally, the soda circulates in the blood, and is not only eliminated in the excess by the kidney, but diffuses itself through the tissues, giving rise to many painful affections due to mechanical irritation. This excess of lithic acid is termed lithæmia. The gouty diathesis would appear to originate in a derangement of that function of the liver by which it disintegrates albuminous foods and converts them into urea.

The problem for us to grapple with is, what are the unfavorable circumstances which cause this diverting of nutritive material from its destination, and throws it off from the economy as useless material, thus robbing the tissues of their needed supply.

What are the circumstances which turn a physiological process into a pathological one?

What are the causes, what the symptoms, what the remedy?

In the answer to the question of causes, I will give, first, an inherited tendency to gouty and rheumatic disease. Prout says: "The children of gouty parents, who have never themselves had gout, are exceedingly liable to lithic acid sediments."

Fothergill and Budd give the same testimony. My own experience has been too limited as yet to form a rule, though I have seen cases which tend to confirm it. After the predisposing are the exciting causes, which are due to an unphysiological life; careless eating both as to quantity and quality. Over-eating throws more material into the blood than can be assimilated, and more than is needed by the tissues for repair, and the excess remains in the blood an irritant foreign substance.

If the total quantity of food is normal, but the carbonaceous in excess, the result is more material than the oxygen can act upon and convert into carbonic acid and water, and an imperfectly oxidized waste accumulates in the blood. "If the nitrogenous or albuminous food is in excess of the oxygen, the oxidation," says Draper, "is arrested at the formation of lithic acid." Whether the cause be too much food, or too small a supply of oxygen, the result is the same. Thus the epicure and the dweller in the overcrowded, ill-ventilated tenement house may suffer from the same cause; an insufficient oxidation of his nitrogenous food resulting in Lithæmia.

As to the symptoms—

Those who have read the literature of Prout, of Garrod, Murchison, Fothergill and others, will say their name is legion. Lithæmia, or a deposit of lithic acid, may occur in so many different tissues that the recognition of the constitutional poison is necessary for a diagnosis.

The liability of the mucous membrane to disease from irritation is too well known for elaboration. It is sufficient, after calling your attention to the fact of a circulating blood supply loaded with a quantity of insoluble lithic acid, to say that many of the symptoms that are hurriedly called dyspeptic, should be called Lithæmic.

As a rule, we find flatulence, costiveness and intestinal colic, alternating with diarrhœa. The tongue is coated, and a little dry. If the deposit is also in the muscular system, as it may be and often is, the symptoms are those of pain, and stiffness, and soreness of the muscles, as in rheumatism. If in the skin, the patient may complain of boils, acne, eczema, erythema, or there may be no rash, but intolerable itching; or else the pins and needle sensation, while visions of impending paralysis make him gloomy and despondent.

There are other and more alarming symptoms which result from this noxious blood supply. The nerve sheaths are inflamed and sciatica and other neuralgiæ result. There are other symptoms which simulate those from severe brain and spinal disease. Prominent among these are vertigo, tinnitus aurium, cramps and muscular twitchings, sleeplessness and irritability.

Dr. Lyman details the following case of a physician in practice twenty-five years, who had occasional attacks of vertigo, accompanied by tinnitus, which was almost constant day and night. He says of him in 1879, eight years after first attack: " A sudden and severe attack occurred one morning with distressing faintness and prostration. For the ensuing two years, there were but slight attacks of vertigo, but the inability to walk straight became manifest. The staggering could only be overcome by stopping, sitting down, or grasping a tree or fence."

This case is given in detail by Dr. Lyman, and is worthy of careful reading, as the man recovered by treatment directed to the lithæmic condition. He duplicates this case with others of equal interest, but which I cannot detail.

DaCosta reports cases of vertigo with irregular action of the heart, of headache, with impaired vision, of neuralgia, dyspepsia, gastralgia, pains in joints of fingers and toes, burning sensations of tongue, palms of hands and soles of feet, pain in the coccyx, and localized anæsthesia.

Of mental conditions, he says: "There are spells of langour and lassitude which befall the man whose blood is charged with lithic acid, in which all exertion is painful. Then there is depression of spirits and gloom, which amount almost to melancholy. But above all is irritability of temper. Odors annoy, sounds infuriate, nothing pleases. The man is on edge; an acid blood literally makes an acid temper. Indeed, many a man who has the reputation of being a curmudgeon, is simply a lithæmic.

Of the mental faculties, the memory is the only one which really suffers. It deteriorates rapidly, especially after lithæmic vertigo, but in time, as the lithæmia is kept away by a careful mode of life, it regains most of its original strength."

I have quoted DaCosta so fully, in order to give the strength of his reputation to a statement of my own cases. Case: An Irishman, of heavy build, consulted me in February, 1884, because of sudden dizzy spells which came on him at irregular times. He made no other complaint; otherwise, he felt well.

Examination of urine found it acid, sp. gr. 1025, reddish color. Bowels moved about three times a week. Tongue furred and moist. Pulse full and 76. Patient was too ignorant to tell whether or not his food distressed him.

The duration of the complaint was about three months. He had been idle since October. This case was instructive to me, and I will give you my reason for making the diagnosis of Lithæmia, its cause and history. The strong, active, hard-working, hard-eating laborer suddenly becomes idle, *i. e.*, he suddenly is deprived of his normal exercise. The needs of the system are lessened, but the nutritive supply is not diminished, and the quantity that is thrown to the tissues is greater than receives complete oxidation, owing to the diminished disassimilation.

The result is the formation of lithic acid, which is manifested by the highly colored urine, and which is the cause of the vertigo. His food consists in the main of fried meat, including pork and boiled potatoes. I prescribed the following treatment: First, a diminished quantity of food and little meat, and mostly vegetable; no liquor. Second, regular exercise, whether he was paid for his work or not. Third, Rochelle salts every morning.

The following :

| | | |
|---|---|---|
| ℞ | Soda, Bi-Carb., - - - | 10 grs. |
| | Potass Nit., - - - - | 20 grs. |
| | Sal. Roch., - - - - | 30 grs. |

in water after meals.

All these the man faithfully carried out, as he was thoroughly frightened. After four weeks, he had no further vertigo. He kept up the alkaline powder, and increased the food. Six months after, he called to tell me he had no return of the fits. This case was a very valuable one, as it was one which, six months before, I should have diagnosed, perhaps, as epilepsy, of petit mal variety. Another case, similar to this one, I saw with Dr. Burwell.

M. S., fifty-three years, a farmer at Hamburg, came to Dr. Burwell, complaining of itching of skin, numbness of fingers, and dizzy spells. He was very costive, bowels moving but once or twice a week, urine scanty, 1027 acid. Dr. Burwell gave him Hunyadi Janos water in the morning, alkaline powder of soda, fifteen grs., nitrate potass, fifteen grs., Rochelle salts, thirty grs., after meals in a quantity of water. He prohibited sugar, meat and liquors, and advised him to eat sparingly at meals. Two months after, he called to say that all unpleasant symptoms had disappeared.

J. D., aged seventy, I saw through the courtesy of Dr. DeLancey Rochester. Patient gave a history of several previous attacks of rheumatism. At present he complained of headache, dizziness, sharp pains through chest, back and legs, flatulence and costiveness. Examination of urine shows it to be strongly acid, one and a half pints in twenty-four hours, and great deposit of urates. He being still under treatment, it is too early to give the result. Cases of urticaria I have taken the liberty to class under the head of acute lithæmia. In dispensary patients, I have found cases of intercostal neuralgia, and various ill-defined pains, on careful questioning, to depend on a lithæmic state of the system. Their frying-pans are the cause of many of their ills. By teaching them what to cook, and how not to cook it, they soon improve. The pathology and etiology being accepted, the treatment should be directed to the removal of the cause. If it be to a diminished supply of oxygen, and your patient is living in close rooms, increase the supply of air if possible. Advise exercise, that respiration may go on more rapidly, and that thus the blood may be oxygenated more thoroughly. If the patient be one whose errors in diet are manifest, restrict him. Let his food be simple. Prohibit meat and fats, pastry, and rich gravies, all fried food and sugars; add to this brisk exercise and skin friction, and your patient has begun to live right.

As medicine, I have been in the habit of giving five grs. of blue pill every night for six nights. Hunyadi Janos water in the morning and the alkaline powder after meals. In the course of two weeks, if the case is not of very long duration, your patient, as a rule, is well enough to be trusted to take care of himself, with the admonition to eat sparingly and keep the bowels free by rigid attention to diet, exercise, and occasional use of the natural mineral waters. A short resume will close he paper. An excess of lithic acid and the lithates in the urine is accounted for by two different theories.

1st. That it represents an arrest of tissue waste at the time it is uric acid before it becomes urea.

2d. That it is the result of the arrest of assimilation of peptones because of an excess of albuminous food over the supply of oxygen.

Whichever theory we accept, we know the original cause is an improper diet and lack of exercise, with hereditary tendency as a predisposing element. We know that the insoluble lithic acid poison may, by acting as an irritant, affect any tissue, and give rise to numerous symptoms, notably those of pain and soreness in the muscular system, itching, perverted sensation, and rash, when the skin is affected; the various neuralgiae by irritation of nerve trunks. There are also the gastro-intestinal symptoms, as costiveness or diarrhœa, flatulence, intestinal colic and dyspepsia, cerebral symptoms of cephalalgia, vertigo, tinnitus aurium, irritability and loss of memory; spinal symptoms of cramps, muscular twitching and unsteady gait.

The treatment is simple and satisfactory, consisting of a diet of fish, vegetables and fruit, avoiding all fried food, fats, sugars and malt liquors, and always eating a moderate quantity at any meal; a regular and vigorous exercise; airy bedrooms. As for medication, it comes last, as being least important; it consists in regulating the bowels by mineral water, and the taking of alkalies, as soda, potash and lithia.

